# Potential of dynamic bacterial communities in the bio-corrosion process: a proof study with surface morphology of metal coupons†

**DOI:** 10.1039/c9ra01959f

**Published:** 2019-05-31

**Authors:** Priyanka Basera, Meeta Lavania, Banwari Lal

**Affiliations:** The Energy and Resources Institute (TERI) IHC Complex 110003 New Delhi India meetal@teri.res.in

## Abstract

Bio-corrosion is a well-known phenomenon of corrosion caused by bacterial communities. It is considered as a worldwide problem as it causes billion-dollar damages to the pipeline industries (mainly oil and gas) each year. Therefore, this investigation was undertaken to understand the significance of bacterial communities in the bio-corrosion system by studying the physical alteration in the metal surface of coupons through different techniques (EIS, XRD, FT-IR and SEM) and the community identification of consortia responsible for the corrosion. Furthermore, supporting data were obtained from APS reductase assays and DAPI microscopy. The EIS plots suggested that the metal coupons in a biotic system were more prone to corrosion than the coupons in an abiotic system. FT-IR analysis of the biotic system validated the presence of magnetite (Fe_3_O_4_), goethite (α-FeOOH) and lepidocrocite (γ-FeOOH); the XRD spectrum confirmed the presence of oxide and sulphide of iron (Fe_3_O_4_ and FeS), which are considered as notable compounds for corroding substances. The community profile indicated the presence of mixed anaerobic consortia containing *Firmicutes* and *Proteobacteria* (*beta* and *delta*) in the cultured sample. The presence of *Desulfovibro sp.* and *Clostridium sp.* in the consortium revealed a synergistic effect, where the by-product of one species acted as a carbon source for the other species, which further established the bio-corrosion process by depositing oxides of iron and sulphur on the metal coupon surface. This study signifies that a mixed culture has a greater impact on the bio-corrosion process than the pure and single culture of *Desulfovibro sp.* Furthermore, this study also provides a bio-monitoring strategy for the pipeline industries.

## Abstract 2

Bio-corrosion is a well-known phenomenon of corrosion caused by bacterial communities.

## Graphical abstract

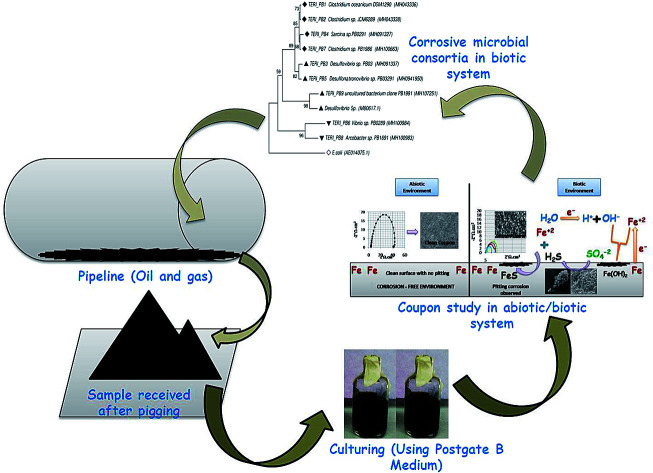

## 1. Introduction

Corrosion study had a significant impact on pipeline industries (especially oil and gas fields); in the past decades, the entire world has been linked through a network of pipelines. They are considered as one of the helpful resources for transporting natural gas, oils, synthesized chemicals and fuels. Transportation through pipelines is considered as the most dependable, safe and easily accessible approach. Although pipelines are protected, incidents can happen, for which corrosion is one of the major explanations and therefore it becomes a key issue.

Financial losses to pipeline industries caused by bio-corrosion are well-documented.^1^ Billions of dollars are spent annually for the replacement of corroded structures and for maintaining their reliability and integrity.^2–4^ According to Zhang *et al.*,^5^ bio-corrosion was considered as one of the possibilities for the leakage in the Alaska oil pipeline at Prudhoe Bay in 2006. Based on the literature, it has been estimated that nearly 20% of corrosion is caused by microbes, which are ubiquitous and diversified in environments such as marine water, oil fields, and cooling power stations.^6^ Furthermore, bio-corrosion is becoming a popular study; it is characterized by the complex processes of different microorganisms (primarily anaerobic) participating in electrochemical reactions and secreting proteins and metabolites, which can have corrosive effects.^3,7^

Corrosion is defined as a complex phenomenon of multiple reactions, where bacteria can use electrons to drive their metabolic pathways.^8^ In addition to this, Enning *et al.*^9^ illustrated that highly corroded metal surfaces could act as semiconductors and help in improving the electrical path for the transfer of electrons to bacteria. Isolated microbial communities from corrosive environments in the oil and gas pipelines account for the presence of sulfate-reducing bacteria (SRB), and they are identified as the principal causative organisms in bio-corrosion.^10,11^ Acetogenic bacteria (*Sporomusa sphaeroides*, *Sporomusa ovate*, *Acetobacterium woodii* and *Acetobacterium carbinolicum*) were also reported to be supportive bacteria for SRB in the bio-corrosion process by producing various organic acids (acetic acid, butyric acid and formic acid).^12–14^ The corrosive nature of SRB is mainly due to the production of H_2_S gas, which is considered as a highly corrosive gas.^2,10,15–17^ It reacts with metal surfaces and is responsible for creating pits, holes and microscopic cracks by generating corrosive products such as FeS.^10^

Although much work has been performed by several researchers in the past decades using various methodologies, it is a subject of great interest to understand the electrochemical complexity behind the bio-corrosion phenomenon, which arises due to the multiple and simultaneous reactions between the pipeline material and the surrounding environment (microorganisms). The present investigation focuses on the leading issue faced by pipeline industries, *i.e.*, bio-corrosion due to anaerobic bacteria with an objective to examine the surface of metal coupons in terms of pitting corrosion and microscopic cracking through proper approaches, *i.e.*, EIS, SEM, XRD, and FT-IR as well as community identification for a better perception towards the synergistic behaviour of bacterial communities in the bio-corrosion process.

## 2. Materials and methods

### 2.1. Coupon preparation

To inspect bio-corrosion, carbon steel metal coupons of dimensions 60 × 10 × 1 mm (length, width and thickness, respectively) were used. The coupons were sterilized using 70% alcohol; furthermore, the sterilized coupons were dried and stored in desiccators (until use). The coupons were incubated for 7, 14, 21 and 28 days at 37 °C in the Postgate B medium (detailed composition is described in Section 2.3). Usually, pigging is conducted for cleaning the pipelines on a quarterly or monthly basis. However, if the conditions are observed to be severe in the pipeline, then, 15 days pigging can be performed. Therefore, in the present research, over a period of one month, weekly (7, 14, 21 and 28 days) data set of coupons was collected.

### 2.2 Sampling site

For the development of microbial consortia, samples were collected from the low gas producing wells in the Mehsana (72.04° E; 23.49° N) Asset of the Oil and Natural Gas Corporation Limited (ONGC) in the western part of India. These reservoirs are situated in a small town, Mehsana, located 75 km away from Ahmedabad, Gujarat, in the Cambay rift valley, which is also known as the intracratonic rift graben. Mehsana has an average elevation of 265 feet (81 m) above the sea level, and the total area of the Mehsana District is 5600 sq. km. The average temperature during the summer months is around 42 °C, while in winter, it is 7 °C. It receives a medium rainfall of 132 cm during monsoons. The groundwater level is about 650–800 ft. Samples were collected from the wellheads of the gas producing wells in the Mehsana Asset. On-site inoculations were performed and transported to TERI for further investigations.

### 2.3 Bacterial culture and establishment of biotic and abiotic systems

To understand the corrosive behaviour of the samples, Postgate B liquid medium was selected (NACE, 2014).^18^ The medium contained (g l^−1^ in de-ionized water) the following solutions: solution 1: 0.5 g, KH_2_PO_4_; 1.0 g, NH_4_Cl; 1.0 g, CaSO_4_; 2.0 g, MgSO_4_·7H_2_O; 25 g, NaCl; 3.5 ml, Sodium lactate (60%); 1.0 g, yeast extract; 0.001 g, resazurin (as an oxygen indicator) at pH 7.5 ± 0.2 (adjusted with 1 M KH_2_PO_4_). Solution 2 (acts as a reducing agent): 0.1 g, ascorbic acid; 0.1 g, thioglycolic acid; 0.001 g, resazurin at pH 7.5 ± 0.2 (adjusted with 4N NaOH) and solution 3: 0.5 g, FeSO_4_·7H_2_O; 0.001 g, resazurin at pH 1.9 ± 0.2 (adjusted with conc. HCl, added dropwise until ferrous sulphate dissolved).

The medium was sparged under an oxygen-free environment by using nitrogen gas, which removed the dissolved oxygen. The medium was dispensed in serum bottles and sealed with butyl rubber stoppers. The three solutions were combined after separately autoclaving (conditions: 121 °C for 15 min). All the inoculated bottles (inoculum was added to 10%) were incubated at 37 °C for 15–28 days. Furthermore, after obtaining 0.5 MacFarland standard turbidity of bacterial growth, which is equivalent to 1.5 × 10^6^ CFU ml^−1^, the coupon study was performed. Sterilized coupons were sealed within the media bottles. Coupons without inoculum (sterilized media only) were considered as abiotic systems, whereas coupons with inoculum were considered as biotic systems and incubated for 7, 14, 21 and 28 days at 37 °C.

All the experiments were performed in triplicates. The data points are the average of the triplicate ± standard deviation (less than 5% of average).

### 2.4 Chemical analysis of bio-corrosion products

#### 2.4.1. X-ray diffraction (XRD)

Analysis of corrosion products formed in the abiotic and biotic systems was performed by employing a RIGAKU (MiniFlex) X-ray diffractometer using Cu-Kα radiation. XRD histograms were obtained from one gram of scraped material (scrapped by a sterilized plastic tool) from the specimens (abiotic and biotic coupons). XRD analysis was executed in the 2*θ* scanning range of 3°–90° with the speed of 4.00 deg min^−1^ in the continuous mode.

#### 2.4.2. Fourier transform infrared spectroscopy (FT-IR)

FT-IR analysis was carried out to identify the functional groups present in the abiotic and biotic systems. Insoluble complexes formed on the corroded specimens were scrapped, collected and subjected to IR studies using FT-IR spectroscopy (Perkin). All spectra were recorded in the transmittance scale with a mid-measuring region of 400–4000 cm^−1^.

### 2.5. Physico-chemical analysis

The physico-chemical properties of the biotic coupons were studied. This analysis included the measurements of different parameters such as hydrogen ion concentration (pH), total dissolved solids (TDS) and electrical conductivity according to the American Petroleum Institute (API) standards as described in S1 (ESI Table S1†). The detection of heavy metal content and cation/anion analysis along with specific profiling of carbon, hydrogen, nitrogen and sulphur (CHNS) were also conducted. CHNS analysis was performed using IS: 1350/American Public Health Association guidelines, as described in ESI Table S1.†^19,20^ All the experiments were performed in triplicate. The data points are the average of the triplicate ± standard deviation (less than 5% of average).

### 2.6. Electrochemical impedance spectroscopy (EIS)

**Fig. 1:**
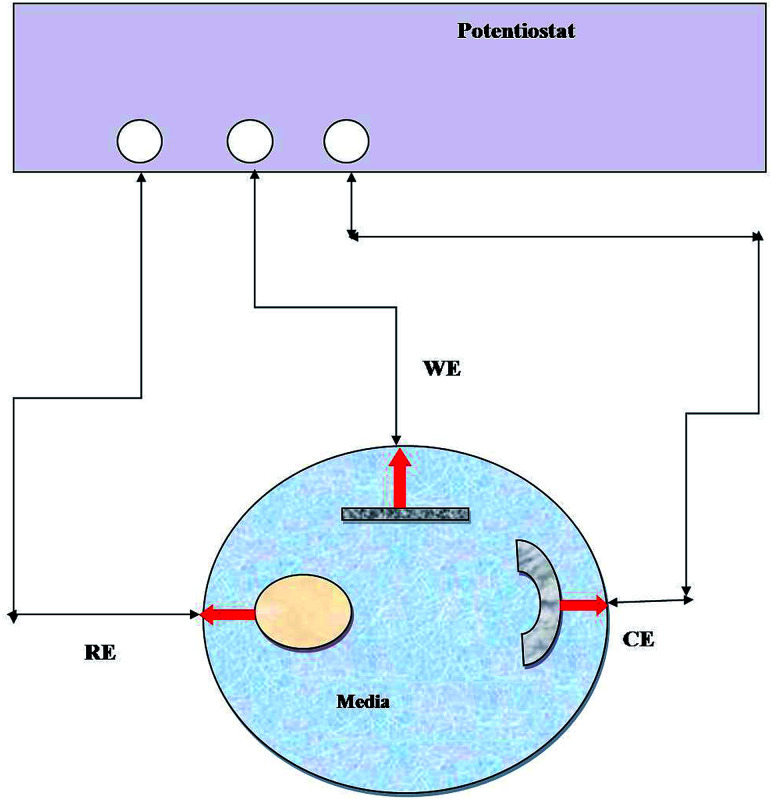
Line diagram of the EIS experimental setup. RE: reference electrode, WE: working electrode, and CE: counter electrode and medium (abiotic and biotic) served as an electrolyte.

EIS has been widely used to measure the electrochemical properties of corroded surfaces. Experiments were conducted in an electrochemical cell containing a three-electrode system: platinum (counter), calomel (reference) and metal coupon (working electrode) (Fig. 1). The working electrodes (metal coupons) were tested in two ways: (i) abiotic (control) and (ii) biotic (treated) systems, where the medium Postgate B acted as the electrolyte. EIS studies were conducted, and data were recorded at 7 days intervals until 28 days of the incubation period. The impedance was represented by the Nyquist plot (imaginary component of impedance *vs.* real component), Bode (phase angle *vs.* frequency) spectra and Tafel plot. EIS was carried out with an AC voltage amplitude of 0.01 V in the frequency range from 0.01 Hz to 10 000 Hz. The data obtained were modelled using the NOVA software.

### 2.7. APS reductase

**Fig. 2:**
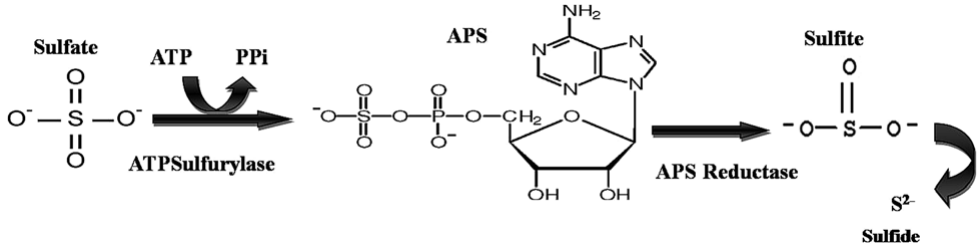
The schematic of the reaction depicts the pathway for sulfate conversion into sulphide, where ATP: adenosine triphosphate, PPi: pyrophosphate and APS: adenosine-5′-phosphosulfate.

The detection of APS reductase (adenosine 5′-phosphosulfate) in the biotic system was performed using the Quickcheck™ SRB kit (as per manufacturer's protocol). The Quickcheck™ SRB Kit was also used for the detection and quantification of SRB. The kit was calibrated with microscopically counted cells of the SRB strain *Desulfovibriode sulfuricans.* APS reductase enzyme is commonly present in sulphate-utilizing organisms and helps in converting sulfates to sulfides, as shown in Fig. 2. The sulfides react with hydrogen and form a corrosive gas (hydrogen sulfide). All the experiments were performed in triplicate. The data points are the average of the triplicate ± standard deviation (less than 5% of average).

### 2.8. Morphological and surface analysis of bacteria and coupons

#### 2.8.1. DAPI (4, 6-diamidino-2-phenylindole) fluorescence microscopy

DAPI is a nucleic-acid specific and well-studied nuclear staining agent. It binds with the minor groove of nucleic acids, specifically in the AT-rich regions. After DAPI fluorescence, bacterial cells appear blue in colour. DAPI was used to inspect the morphologies and count the bacteria present in the biotic system. Samples were subjected to microscopy, and image analysis was executed using an AxioCam MRC Zeiss Microscope. A Zeiss filter set at 03 (excitation 358 nm, emission 461 nm) was used for examining DAPI fluorescence.

#### 2.8.2. Scanning electron microscopy (SEM)

For the surface morphology studies of the abiotic and biotic systems, SEM analysis was performed. Under aseptic conditions, the metal coupons were coated with a gold film to provide conductivity for SEM observations. A Carl Zeiss scanning electron microscope was used to observe the bio-corrosion phenomenon on the metal coupon surface.^21^

### 2.9. Community identification

Microbial communities responsible for corrosive behaviour were identified by universal bacterial 16S rRNA gene sequencing and phylogenetic analysis.

#### 2.9.1. DNA extraction

First, the bead beating procedure was applied to selected samples for 1 min.^22^ Furthermore, the genomic DNA was extracted from the pellet using the PowerSoil DNA isolation kit (MoBio) by following the manufacturer's instructions.

#### 2.9.2. 16S rRNA gene sequencing and phylogenetic analysis

Amplification was conducted using the universal bacterial 16S rRNA gene polymerase chain reaction (PCR) with forward primer 27F-AGAGTTTGATCATGGCTCAG and reverse primer 1492R-GGTTACCTTGTTACGACTT. PCR, cloning and transformation were carried out as described by Lavania *et al.*^23^ The obtained sequences (16S rRNA) were compared to database *via* Basic Local Alignment Search Tool (BLAST) and deposited in GenBank. A phylogenetic tree was constructed by the neighbor-joining method, where 1000 bootstrap replications were carried out to validate the internal branches using MEGA version 6.06 programs.^24^

## 3. Results and discussion

### 3.1. Chemical analysis of bio-corrosion products

**Fig. 3:**
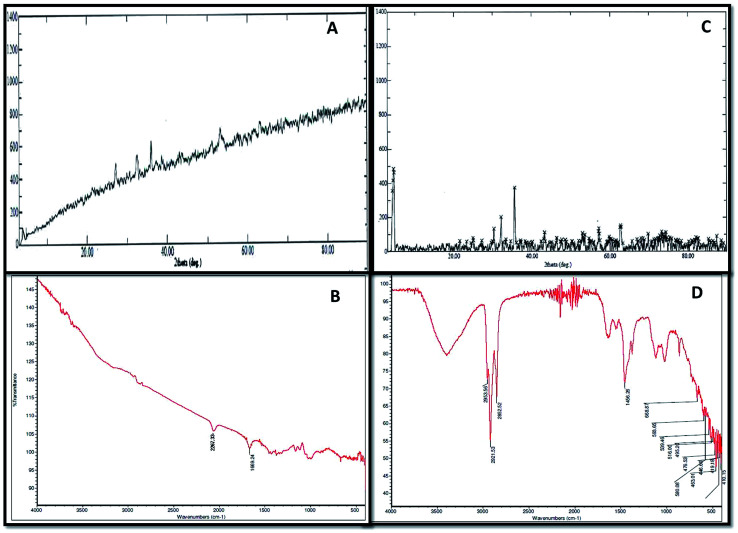
The corrosive chemical composition produced in the abiotic and biotic systems illustrated by FT-IR and XRD spectra. (A and B) Represent the FT-IR and XRD results for the abiotic system, respectively. (C and D) Show the FT-IR and XRD results for the biotic system, respectively.

XRD and FTIR analyses provided information regarding the chemical composition and functional groups (oxides, sulphides and stretching of compounds) of the corrosive products deposited in the abiotic and biotic systems on the metal surface Fig. 3(A–D). The intensity of peaks in the abiotic system (control) was observed to be weak (Fig. 3A). The XRD spectra of the biotic system confirmed the presence of oxide and sulphide of iron (Fe_3_O_4_ and FeS) with high-intensity peaks; the pattern confirmed that the production of sulfide was biogenic in nature (Fig. 3C). Sulfate-reducing bacteria utilized the iron and sulfate (terminal electron acceptors) from the medium and finally altered them to iron sulfide (Fe_*x*_S_*y*_), which is considered as one of the most corrosive agents for pipelines.^16,25^ Similar spectra were reported by Parthipan *et al.*^4^ and AlAbbas *et al.*^22^ The XRD data revealed the role of bacteria in mineral deposition on the metal surface, which were responsible for coupon corrosion.

As such, no major peaks were observed in the FTIR spectra of the abiotic system (control) (Fig. 3B). Only minor peaks at 1667–2300 cm^−1^, which corresponded to the –C=C conjugated dienes and aliphatic groups, were noticed. Furthermore, the FT-IR spectra of the biotic system demonstrated vibrations ranging between 400 and 700 cm^−1^, accounting for the Fe–O stretching, which confirmed the presence of goethite (α-FeOOH) and lepidocrocite (γ-FeOOH) (Fig. 3D). The presence of magnetite (Fe_3_O_4_) in the sample was shown by the peak at 580 cm^−1^. The stretching at higher wavenumbers signified the presence of OH,^26^ whereas the stretching observed between 2500 and 3000 cm^−1^ was assigned to C–H due to the polysaccharide bonds of sugar present in the bacterial biofilm. These compounds are indicated as the notable compounds for corroding a substance. The nature of stretching was described by Kumar and Balasubramaniam; they revealed the presence of similar compounds in Delhi iron pillar rust.^27,28^ The data of the biotic system obtained from XRD and FT-IR analyses indicated the presence of these compounds in major amounts, which are corrosive to their respective environments and play major roles in bio-corrosion.

Table S1† confirms the presence of low pH, high conductivity and heavy metals (iron, sulphate and chloride) in the biotic system. According to AlAbbas *et al.*,^29^ SRB are responsible for producing hydrogen sulfide gas (H_2_S), which acts as an acid gas. The presence of acid gas creates an acidic environment. As reported by Dong *et al.* and Sharma *et al.*,^30,31^ low pH (acidic environments) and high conductivity are responsible for acid attack on metals and create an environment for accelerated microbial corrosion. These results communicate the corrosion mechanism on the coupon bottles.

### 3.2. Electrical impedance spectroscopy (EIS) results

**Fig. 4:**
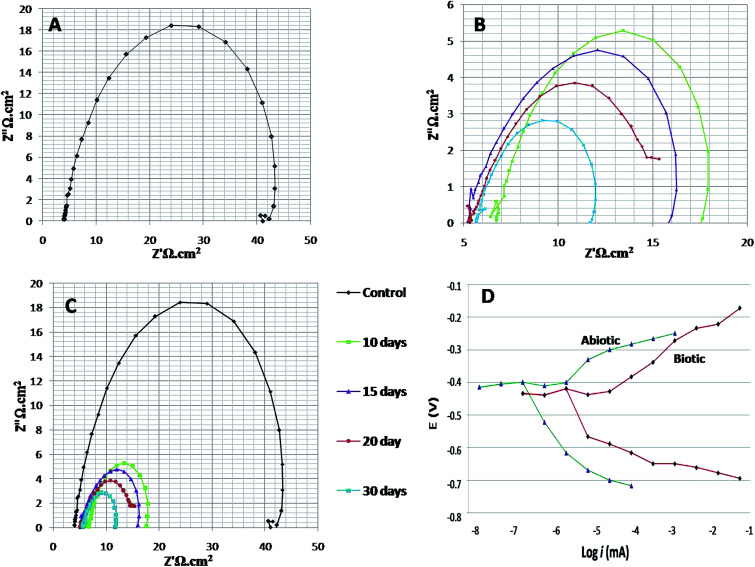
The Nyquist and Tafel spectra. (A) Illustrates the spectra of the abiotic system, (B) illustrates the spectra of the biotic system for 7^th^, 14^th^, 21^st^ and 28^th^ days, (C) shows the combined spectra for both abiotic and biotic systems, and (D) elucidates the Tafel plots of the abiotic and biotic systems (on the 28^th^ day).

**Fig. 5:**
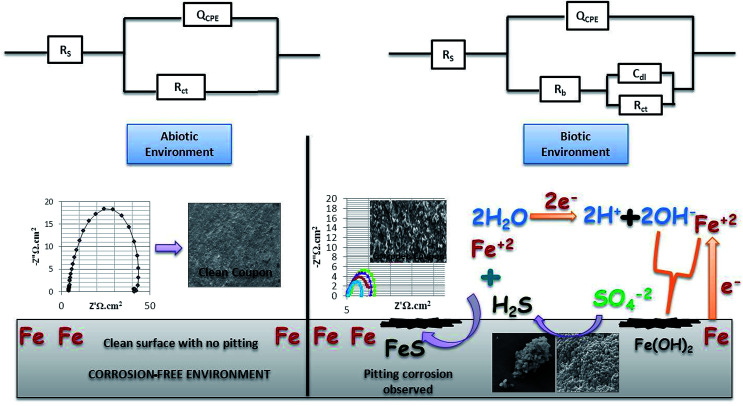
The bio-electrochemical reactions of the abiotic and biotic systems. The biotic system is a potentially active system.

To study the behaviour of microorganisms in the corrosion field, the EIS technique is widely used. In the present research on bio-corrosion, metal specimens were analysed by obtaining the EIS graphs: Nyquist plots, Tafel curves and Bode plots (Fig. 4 and 5). A Nyquist graph is a complex plane of the Cartesian coordinates, where the abscissa is the real part (Z′) and the ordinate is the imaginary part (Z′′); the semicircle diameter represents the charge transfer resistance (*R*_ct_), which is equivalent to the polarization resistance (*R*_p_).^16,32^ To obtained the Nyquist semicircle, parallel connection of ohmic resistor (*R*) and *Q*_CPE_ constant phase element (CPE) was used for the measurement.^33^ To represent the deviation from ideal behaviour, CPE was used instead of a capacitor. Using the following equation, the impedance of CPE can be defined:^22,34^*Z*_CPE_ = 1/*Y*_0_ (*jω*)^*n*^

Here, *j* is the imaginary number, *ω* = (2π*f*) is the angular frequency in radians/sec, *n* = *a* dimensionless parameter that lies between 0 and 1, and *Y*_0_ is a factor related to capacitance. As the diameter of the semicircle increases, *R*_ct_ will increase, which further decreases the corrosion rate.^21^

EIS was recorded on every 7^th^ day of the 28 days incubation period. The diameter of *R*_ct_ was observed to be maximum for control (Fig. 4A); as the environment was abiotic, there was no bacterial activity and the electron transfer was very low. Therefore, in the abiotic system, the chance of electro–biochemical reactions was negligible.

By studying the Nyquist loops of the biotic system, the corrosive nature of the sample can be justified. A significant difference was observed on comparing the diameters of the biotic and abiotic systems; a relatively reduced *R*_ct_ value was observed in the biotic system. The reduced diameter signified the role of bacterial activity in metal corrosion.^35^ After exposing the coupons for different exposure times, it was observed that, as the incubation period increased, the corrosive behaviour caused by bacteria also increased. The diameter obtained on the 28^th^ day was comparatively smaller than the diameter obtained on the 7^th^ day of incubation (Fig. 4B and C). Fig. 4D illustrates the Tafel plots of the abiotic and biotic systems, corresponding to the occurrence of anodic and cathodic reactions, and the intersection in the graph gives the corrosion exchange current density. In the case of the biotic graph, the anodic and cathodic values are higher in comparison to the observations for the abiotic graph. This showed that the biotic system contained a corrosive environment. Study reported by Jia *et al.*,^36^ similar Tafel plots were obtained; the abiotic system showed lesser corrosion values than the biotic system.

Fig. 5 shows the difference in the bio-electrochemistry of abiotic and biotic systems with the circuit model, where *R*_s_ denotes the resistance of the solution, *R*_b_ is the resistance created by the biofilm formed in the biotic system, and *C*_dl_ refers to the double layer capacitance. In the biotic system, the microorganisms accelerated the corrosion process by generating corrosive products on the metal coupons. The reciprocal of charge transfer resistance (1/*R*_ct_) is known as the corrosion rate; the smaller the *R*_ct_, the higher the rate of corrosion. It is evident that the diameter of the biotic semicircle decreases, which further reduces the polarization resistance. This suggests that the biotic system has a noticeable effect on corrosion growth by altering the surrounding environment; the metabolites produced (oxides of iron and sulfur) in the biotic system alter the electrochemical properties of the system, which further results in reduced polarization resistance.^37^

**Fig. 6:**
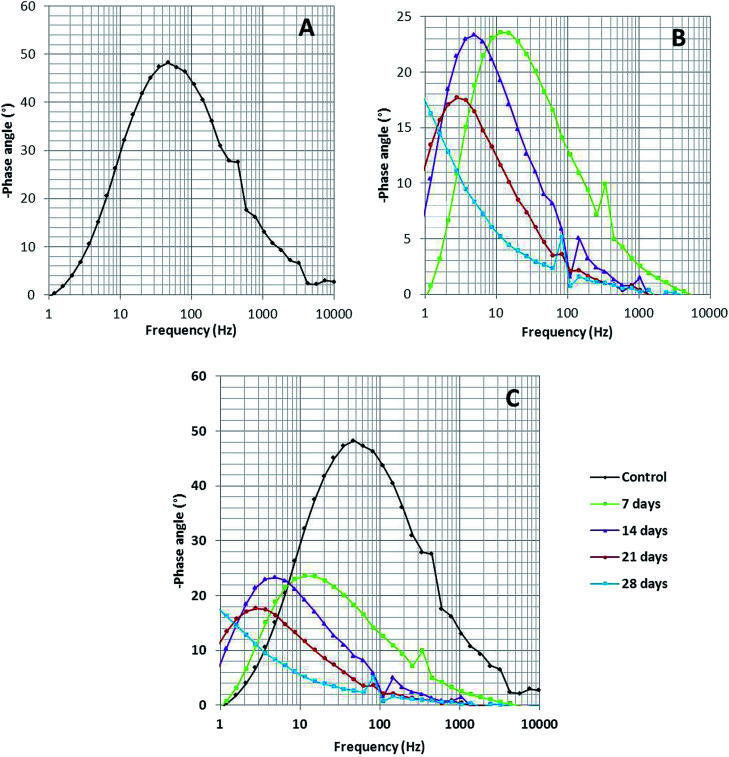
The Bode phase spectra of (A) the abiotic system and (B) the biotic system on 7^th^, 14^th^, 21^st^ and 28^th^ days; (C) depicts the comparison of the abiotic and biotic systems.

Bode spectra (Fig. 6) are plotted in the orthogonal axis planes, in which the phase angle (degree) is on the ordinate axis and the logarithm of the frequency (Hertz) is on the abscissa axis. The phase angle spectrum of the biotic system is smaller than that of the abiotic system, which further represents that the conductivity of the solution sample is high as compared to that of the control.^38^ The higher conductivity clarified the presence of more free ions in the system, due to which the bacterial electric pathways become active and this will speed up the process of corrosion.

For better understanding, combined 3-D visualization of the Nyquist and Bode graphs is provided in Fig. S1† (the abiotic system is represented by black colour and the biotic system by red colour); the area covered (diameter of semicircle) by the abiotic system was much wider than that covered by the biotic system, which imparted a clear picture of the corrosive nature of the biotic system.

### 3.3. Adenosine-5′-phosphosulfate (APS) reductase assay

**Fig. 7:**
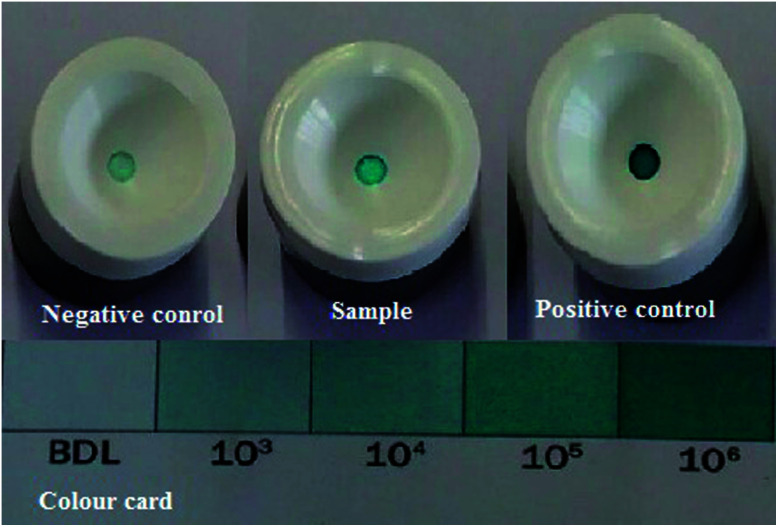
APS Reductase test. The negative control falls in the BDL range (Below Detection Limit), whereas the positive control and the sample show positive responses towards the reaction. All the experiments were performed in triplicate. The data points are the average of the triplicate ± standard deviation (less than 5% of average).

The biotic sample was subjected to the APS reductase assay, which involved a 15 minutes reaction. This assay indicated the presence of APS reductase enzyme in the sample. The kit contained an antibody against the enzyme adenosine-5′-phosphosulfate (APS) reductase, which is common to all strains of SRB. The detection of the enzyme can be examined by the appearance of blue colour on the membrane device after the addition of a chromogen (provided with the kit). Darker shades of blue signify the presence of a higher count of SRB in the sample, which can be inspected by matching the colour card (as per the manufacturer guidelines).^18^ For the present analysis, positive and negative controls were prepared. The negative control fell into BDL range (Below Detection Limit), whereas the sample showed a positive reponse towards the reaction with 10^5^ ± 1.5 cell per mL and the positive control showed 10^6^ ± 1.5 cell per mL bacterial count, as depicted in Fig. 7.

Krumholz *et al.* (2013)^39^ described that *Desulfovibro sp.* used sulfate and converted it to adenosyl phosphor sulphate, which involved ATP and assistance from the enzyme sulfate adenylyl transferase (common in all SRB). Furthermore, APS reductase acted as the catalyst and helped in the reduction of APS to sulfite and adenosine monophosphate. The production of hydrogen sulfide (H_2_S) is a prominent step in accelerating the rate of bio-corrosion in pipeline industries.^11,14^ This data suggest that the community present in the biotic system has the potential for developing bio-corrosion.

### 3.4. Identification of corrosive consortium

**Fig. 8:**
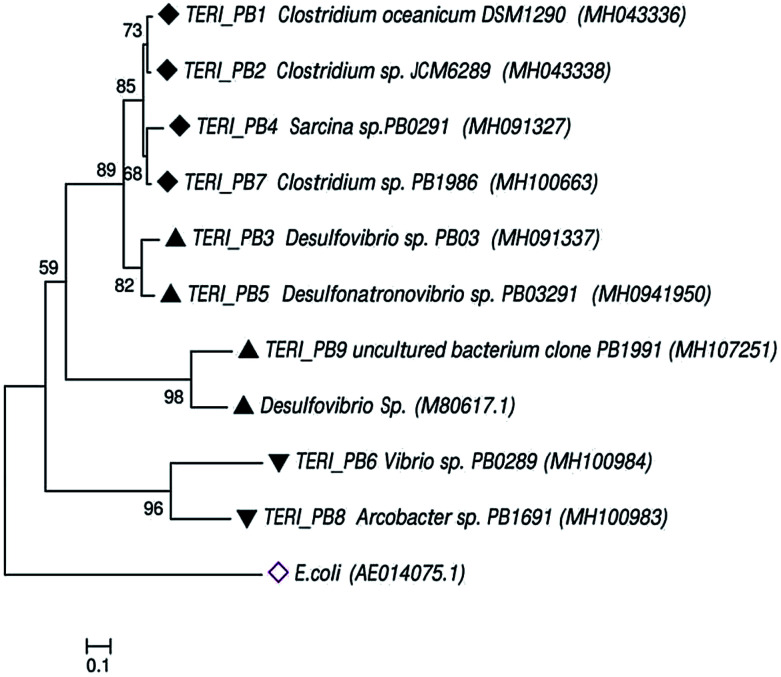
The phylogenetic tree of the consortium was constructed using neighbor-joining through bacterial 16S rRNA gene sequences. The tree shows the relationship within the bacterial domain, *i.e.*, *Firmicutes* and *Proteobacteria*. Numbers at nodes indicate the bootstrap values >50% from 1000 replicates. GenBank accession numbers are provided in brackets. The scale bar indicates sequence divergence.

Uncultured strains were characterized based on 16S rRNA and studied with phylogenetic tree, as shown in Fig. 8. These are available under the accession numbers MH100663, MH100983, MH100984, MH043336, MH043338, MH091327, MH091337 and MH094195. The phylogenetic profile indicated that the bacterial consortium contained mixed phyla of anaerobic *Firmicutes* and *Proteobacteria* (*beta* and *delta*). In the *Firmicutes* phylum, *Clostridium sp.* and *Sarcina sp.* were obtained, whereas the *beta-Proteobacteria* comprised *Vibrio sp.* and *Arcobacter sp.* Furthermore, *delta-Proteobacteria* included *Desulfovibrio sp.*, which are considered as the most prominent hostile phyla for pipeline sectors.^2,9,40,41^

Previous reports suggest that *Desulfovibro sp.* from *Proteobacteria* can be considered as one of the dominant corrosive species.^42,43^ The classical theory of anaerobic bio-corrosion illustrates that energy metabolism, which occurs in the periplasmic space of *Desulfovibro sp.*, uses the electrons from hydrogen, organic acids or alcohols for sulfate reduction. In the case of hydrogen, the hydrogenase enzyme utilizes the electrons from hydrogen, which are accumulated on the surface of the metal. Furthermore, in the cytoplasm, the process of dissimilatory sulfate reduction takes place, where sulfate (as a terminal electron acceptor) is converted into sulfide (a corrosive compound).^8,44^ Vigneron *et al.*^45^ reported the presence of *Desulfovibro sp.* in a corrosive biofilm from the offshore pipelines and considered it as a major reason for bio-corrosion. Enning *et al.*^9^ explained that *Desulfovibro sp.* accelerated the process of corrosion by 70 to 90 times. Acetogenic bacteria such as the *Clostridium sp.* of *Firmicutes* are also an obvious contributor towards bio-corrosion.^46^ Acetic, butyric and formic acids are the common acids produced by *Clostridium sp.* and thus, they have a tendency to promote corrosion in the pipeline sector.^12^

**Fig. 9:**
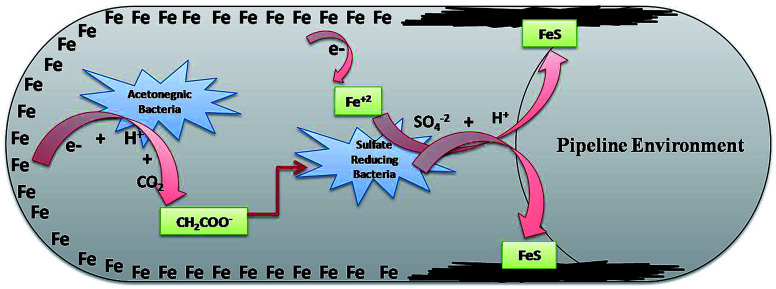
The synergestic effect of the anaerobic bacterial community, where the by-product of one species acts as a carbon source for the other existing species.

SRB are the eminent anaerobic bacterial domains for establishing detrimental aspects in pipeline industries.^45,47^ Bio-corrosion in the pipeline sector is considered as a complex process of multiple reactions. Fig. 9 depicts the line diagram of diverse bacterial communities exacerbating the corrosion process by releasing their metabolic by-products.^2^ The pipeline environment acts as a favourable atmosphere for the metabolic growth of bacteria. It contains hydrocarbons and moisture, which are suitable conditions for bacteria to proliferate; they show synergistic behaviour with other bacterial communities, where the by-products (small volatile fatty acids) of one species act as the carbon source for other species.^5,13,48^ As reported by Ilhan-Sungur *et al.*,^49^*Clostridium sp.* can show a corrosive effect with *Desulfovibro sp.* by providing the electron donor “hydrogen” to them. In this way, they support each other and accelerate the process of bio-corrosion. The data obtained from this analysis indicated that a mixed culture has a great potential towards bio-corrosion.

### 3.5. Morphological and surface analysis of bacteria and coupons

**Fig. 10:**
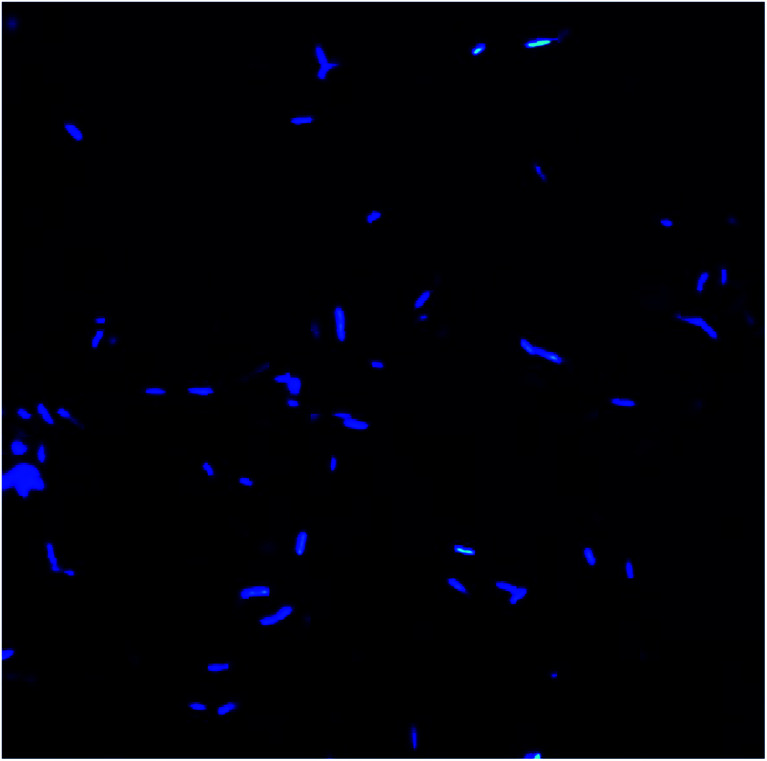
Bacterial morphology under a DAPI filter, which appear to be bright blue rod-shaped with diplobacilli arrangement.

**Fig. 11:**
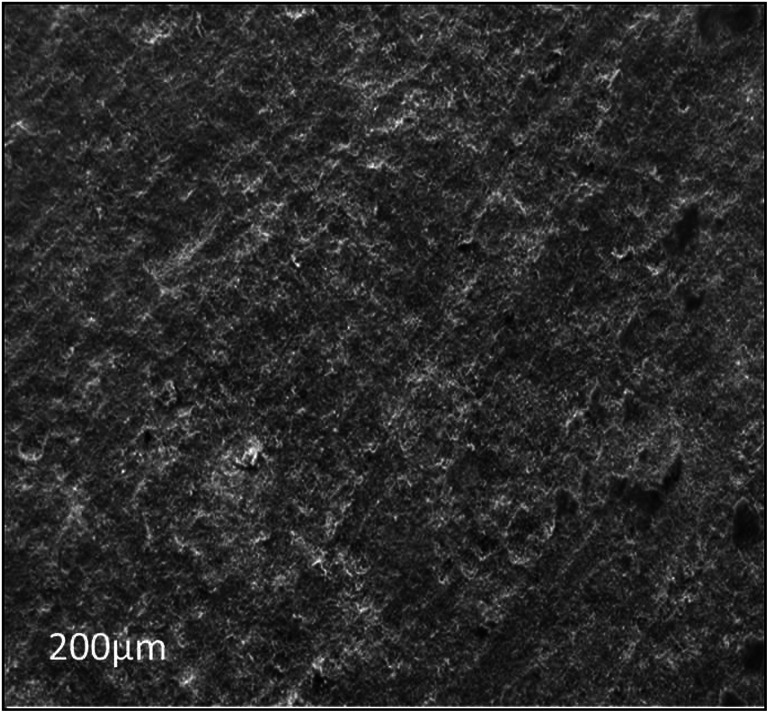
The SEM image of the abiotic system, showing that the metal coupon surface is cleaned after the 28^th^ day of incubation.

**Fig. 12:**
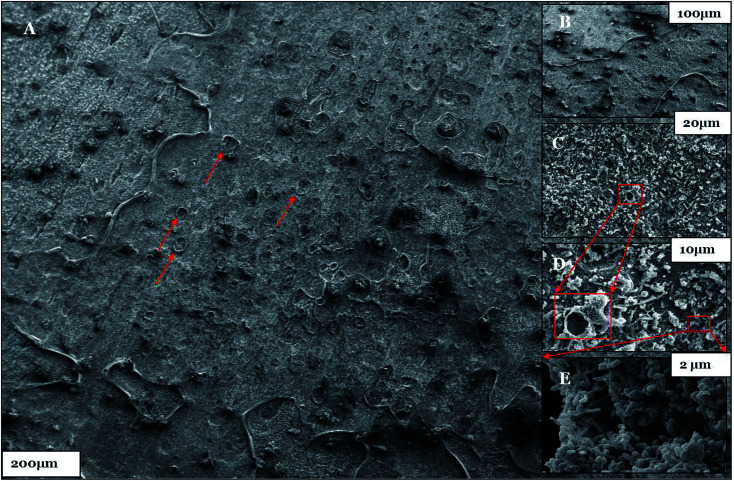
(A–E) The SEM micrographs of the biotic system after the 28^th^ day of incubation. Pit holes are shown by the red arrows and boxes.

**Fig. 13:**
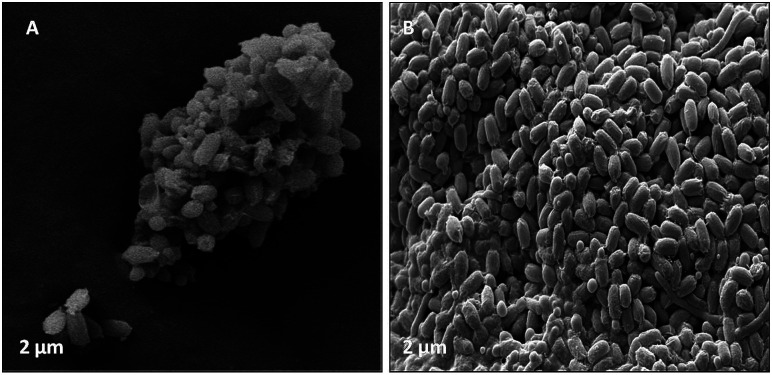
(A) Illustrated the bacterial species had a cumulative effect on bio-corrosion. (B) Demonstrated the morphology (rod shape) of bacterial community associated with bio-corrosion.

Morphology and surface analyses were performed by using DAPI and SEM (Fig. 10–13). Fluorescent dyes can be used to stain microorganisms from virtually any microbial habitat. Therefore, DAPI is a popular stain for this purpose as it targets the DNA of all bacterial cells, *i.e.*, live, dormant and dead. For the detection and monitoring of microorganisms in the pipeline industries, DAPI fluorescence is a fast and easy method.^50,51^Fig. 10 illustrates the bacterial counts in the samples, which are in the range of 10^5^–10^6^ ± 0.5 cells per mL, with rod-shaped morphology. Although there is no well-defined reported threshold value of microbial count, increased levels of corrosive bacterial count might create a threat to the pipeline industries for bio-corrosion.

Furthermore, the level of the corrosive nature of samples was indicated by the scanning electron micrographs of the biotic coupons (Fig. 11–13). Fig. 11 represents the nature of the abiotic system (coupons in a sterile medium), whereas Fig. 12 and 13 illustrate the effect of the biotic system on the metal surface and the bacterial community responsible for corrosion. With respect to appearance, a significant difference was observed while comparing the coupons of the abiotic and biotic systems. The coupons in the abiotic system appeared clean and clear as compared to the coupons in the biotic system. In the biotic system, the bacterial community almost fully covered the coupon surface. While examining the images in Fig. 12(A–E), microscopic cracks and pits can be noticed, as indicated by the red arrows and boxes. Previous studies illustrated that corrosive bacterial species corroded the surfaces of the coupons by producing large amounts of corrosion products mainly composed of iron oxide and an extracellular polymeric substance (EPS). Bacterial EPS mostly comprises polysaccharides, proteins, nucleic acids, phospholipids and humic substances. It supports the bacteria for holding on to the metal surface and is thought to play a significant role in the corrosion process.^5,52,53^

Fig. 13 illustrates that bacteria work in a cumulative way to accelerate corrosion in the surrounding environment. By accumulating, they reduce the surface area with respect to the environment.^54^ The possible reason for the pits could be elucidated by associating the data obtained from EIS, XRD, and FTIR as well as the APS reductase assay and community analysis, which provide evidence that the consortium contains corrosive anaerobic bacterial species and assists the process of corrosion synergistically.^55^

## 4. Conclusions

The present research focused on the corrosion of pipelines mediated by multiple bacterial communities. This study also reported that valuable techniques (EIS, XRD, FTIR and SEM) can be correlated together for developing a better understanding of bio-corrosion. The results illustrated that bio-corrosion is a synergistic behaviour of dynamic microorganisms belonging to *Firmicutes* and *Proteobacteria* (*beta* and *delta*). With the help of the coupon study, it was elucidated that the corrosion process was accelerated in the biotic system. The findings of the present work establish a strategy for bio-monitoring in pipeline industries.

## Conflicts of interest

There are no conflicts to declare.

## Supplementary Material

RA-009-C9RA01959F-s001
