# Supplementary material for: Potential of dynamic bacterial communities in the bio-corrosion process: a proof study with surface morphology of metal coupons
Source: RSC Adv. 2019 May 31;9(30):17040–50. doi: 10.1039/c9ra01959f (PMC9064554; doi:10.1039/c9ra01959f)
Supplement: RA-009-C9RA01959F-s001 [file RA-009-C9RA01959F-s001.pdf]

Electronic Supplementary Material (ESI) for RSC Advances.
This journal is © The Royal Society of Chemistry 2019

Supplementary file

Table S1: Physico-chemical analysis of biotic sample

**Test parameters**	**Methods**	**Observation**
pH	EPA 9040 C	6.34 ±0.20
Conductivity µS/cm	EPA 9050 A	43.4 ±0.12
TDS (ppm)	APHA, 1998	2.78×10^{7} ±0.15
**Cation (mg/l) /Anion (mg/l)**		
Chloride	IS:1760(P-5)	4423.6 ±20.0
Sulfate	EPA-9038-1	195.6 ±1.80
Sulfide	APHA 4500-S 2- F :2012	5.0 ±0.10
Aluminum	APHA3111(B)	145 ±2.20
Cobalt	APHA3111(B)	0.50 ±0.09
Chromium	EPA SW 846-7190	<0.01 ±0.00
Copper	EPA SW 846-7210	11.20 ±0.09
**Elemental Analysis (%)**		
Iron	IS:1527-1972	47.82 ±0.9
Aluminum	IS:1527-1972	4.56 ±0.01
Sulfur	Is:1350(P-3)	2.75 ±0.05
**CHNS Analysis (%)**		
Carbon	IS:1760(P-5)	46.81 ±0.50
Hydrogen	IS:1350(P-4/sec1)	4.21 ±0.02
Nitrogen	IS:1350(P-4/sec2)	2.10 ±0.01
Sulfur	IS:1350(P-3)	2.72 ±0.01

All the listed testing's performed in triplicate. The data points are average of the triplicate ± standard deviation (less than 5% of average).

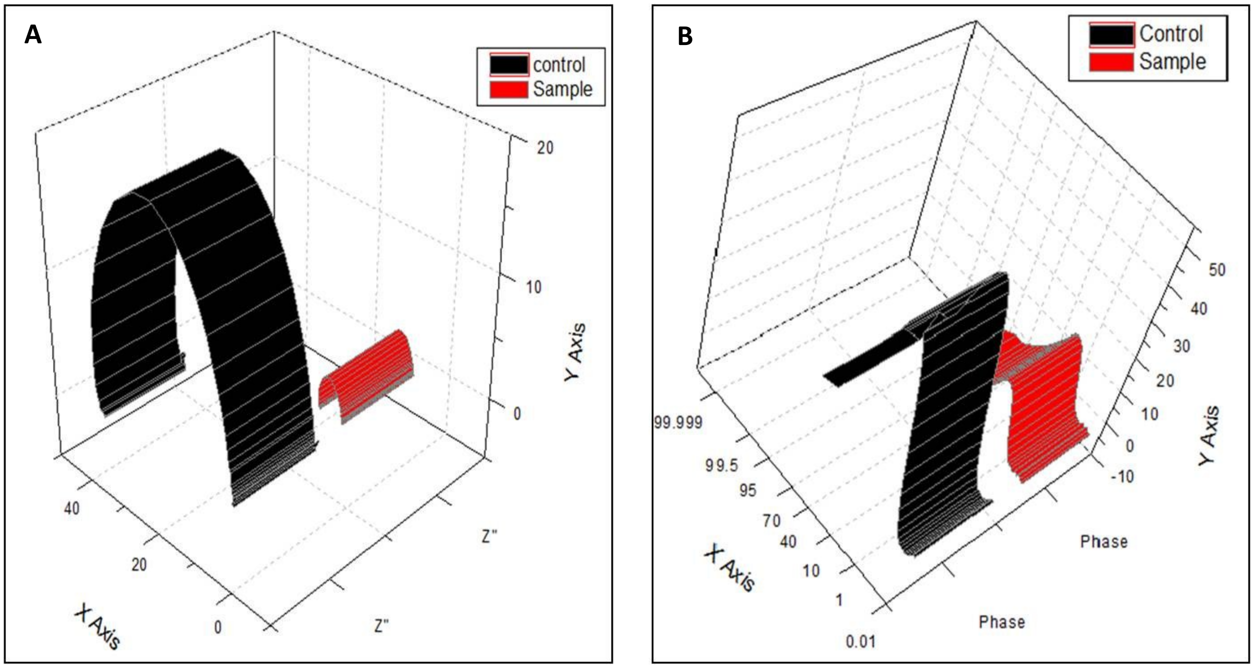

Fig .S1. (A) 3D spectra of Nyquist plot depicted the area covered by biotic system is much smaller than the area covered by abiotic system (B) 3D spectra of Bode plot illustrated the peak of biotic system is smaller than abiotic system.
